# Kinetic ^15^N-isotope effects on algal growth

**DOI:** 10.1038/srep44181

**Published:** 2017-03-10

**Authors:** Eivydas Andriukonis, Elena Gorokhova

**Affiliations:** 1 Faculty of Chemistry and Geosciences, Department of Physical Chemistry, Vilnius University, Vilnius, Lithuania; 2 Laboratory of Bio-Nanotechnology, Center for Physical Sciences and Technology, Vilnius, Lithuania; 3 Department of Environmental Science and Analytical Chemistry, Stockholm University, Stockholm, Sweden

## Abstract

Stable isotope labeling is a standard technique for tracing material transfer in molecular, ecological and biogeochemical studies. The main assumption in this approach is that the enrichment with a heavy isotope has no effect on the organism metabolism and growth, which is not consistent with current theoretical and empirical knowledge on kinetic isotope effects. Here, we demonstrate profound changes in growth dynamics of the green alga *Raphidocelis subcapitata* grown in ^15^N-enriched media. With increasing ^15^N concentration (0.37 to 50 at%), the lag phase increased, whereas maximal growth rate and total yield decreased; moreover, there was a negative relationship between the growth and the lag phase across the treatments. The latter suggests that a trade-off between growth rate and the ability to adapt to the high ^15^N environment may exist. Remarkably, the lag-phase response at 3.5 at% ^15^N was the shortest and deviated from the overall trend, thus providing partial support to the recently proposed Isotopic Resonance hypothesis, which predicts that certain isotopic composition is particularly favorable for living organisms. These findings confirm the occurrence of KIE in isotopically enriched algae and underline the importance of considering these effects when using stable isotope labeling in field and experimental studies.

Application of stable isotope labeling techniques in life sciences is growing, particularly in molecular and biochemical studies, but also in human and environmental health, agriculture, biogeochemistry, ecology, and ecotoxicology. In ecology and ecophysiology, both natural abundances of ^13^C and ^15^N and sources artificially enriched in these isotopes are used for tracing material transfer in material flows and biogeochemical processes^1^. In systems biology, isotopic labeling is increasingly applied for proteomics and metabolomics analyses to better understand the organization and the functioning of metabolic systems^2^. For example, SILAC (stable isotope labeling by/with amino acids in cell culture) technique based on mass spectrometry detects differences in protein abundance among samples using isotopic labeling. This technique allows studying cell signaling, post-translation modifications and regulation of gene expression^3^, and changes in these processes caused by environmental stress and disease^2^,^4^. In metabolomics, stable-isotope labeled biomolecules (sugars, fatty acids, and amino acids) are employed to model metabolic flux or biochemical pathways by tracking isotope distribution through various molecules^4^.

Stable isotope markers are the heavy isotopes that contribute only a minor part of the respective elements: ^2^H (deuterium), ^13^C, ^15^N, ^18^O, and ^34^S. As these elements occur in nearly any biomolecule, replacing light isotopes with their heavier counterparts would provide a label to trace these biomolecules and their products. However, the main assumption here is that the increased levels of a heavy isotope have no effect on the organism/cell growth and metabolism; i.e., the introduced isotopes do not modify the operation of the metabolic pathways, and the quantity and quality of the synthesized material are independent of the isotopic composition of the isotopic milieu.

Kinetic isotope effect (KIE) is the change in the rate of a chemical reaction when one of the atoms in the reactants is replaced with its isotope. Heavier isotopes form stronger bonds that require higher energy to break them, which ultimately slows down the chemical reaction rate^9^,^10^. Slower reactions for organisms, in the context of isotopic labeling, can result in changing metabolic fluxes^11^ and decreased growth and development^7^,^12^. A few studies have addressed effects of externally supplied heavy isotopes on the growth and morphology of algae. Cumulative enrichment of H, C, O and N with respective heavy isotopes resulted in a changed morphology and slower growth of the green alga *Chlorella vulgaris*^13^, with strongest alterations attributed to enrichment with ^2^H and ^18^O isotopes. In these studies, however, the focus was on algal cell size distribution, protein and nucleic acid content^13^, while growth kinetics has not been analyzed. The full isotope labeling of autotrophic organisms for nuclear magnetic resonance spectroscopy (NMR) and mass spectrometry gained popularity since ^13^C and ^15^N isotopes were not found to affect algal growth measurably^14^.

The evidence is accumulating, however, that KIEs occur in bacteria, fungi, and yeast growing in media enriched with a heavy isotope^7^,^8^,^15^,^16^. Recent applications of isotope labeling in metabolomics indicate that many enzymes may be susceptible to these effects^5^, and microorganism growth changes with increasing proportion of heavy isotopes in the media^6^,^7^,^8^. The observed alterations in microorganisms exposed to isotopically enriched media include a prolonged lag phase and slower growth rate, but also increased morphological aberrations, longevity and decreased oxidative stress^16^. It has also been suggested that ^15^N generates the lowest KIEs compared to ^13^C and, particularly, ^2^H and ^18^O^13^,^17^.

Another phenomenon related to KIEs is a non-monotonous growth response to the increasing concentration of heavy isotope in the growth medium. In particular, according to the Isotopic Resonance (IsoRes) hypothesis, growth is favored at certain isotopic compositions^18^. The IsoRes hypothesis and its predictions are based on the analysis of mass using two-dimensional (2D) plot for monoisotopic mass defect (NMD) and a normalized isotopic shift (NIS) in peptides at different isotopic ratios of C, H, O and N as the horizontal and vertical axes, respectively^19^. Due to the facts that elemental composition of biomolecules has a discrete character and that molecules have isotopic distribution (just like elements), the monoisotopic defect and isotopic shift are also discrete. When an isotopic resonance occurs, a straight line corresponding to a strong and significant correlation between NMD and NIS becomes apparent, with the fraction of the molecules on the line determining the relative strength of the resonance^18^,^19^. The resonance conditions are characterized by an overall reduction of the system complexity, viewed as a total number of distinct quantum mechanical states. In general, the reduced complexity is likely to increase the kinetics of chemical and biochemical reactions, thus maximizing synthesis of amino acids and growth. The isotopic resonance occurs at many different combinations of isotopic abundances for H, C, N and O, the main elements of living cells. In particular, nitrogen exhibits resonance conditions at 0.37, 3.5 and 10.9 at% ^15^N^17^. The experimental support for this hypothesis was provided by experiments with *E. coli* grown at varying isotope composition of H, C, N, and O^17^.

It is crucial to understand KIEs in various living systems if we are to apply stable isotope labeling technique in ecological, biogeochemical, physiological and molecular studies. Neglecting these effects and resonance conditions may result in creating artificial systems that do not reflect true *in situ* responses. Here, we explored KIE of ^15^N in green algae, eukaryotic autotrophs, by conducting a standard growth inhibition assay in at% ^15^N concentrations ranging from 0.37% (natural abundance) to 50% (heavily enriched). Based on the earlier reported KIEs in bacteria^20^ and algae^21^, we expected that altered ^15^N concentration would decrease growth performance of the algae adapted to the terrestrial isotopic composition. We also hypothesized that this growth depression would not occur for the algae exposed to 3.5 at% ^15^N because this concentration provides resonance conditions favorable for growth.

## Materials and Methods

### Test organisms

The freshwater green alga *Raphidocelis subcapitata* (Korshikov) Nygaard, Komárek, J. Kristiansen & O. M. Skulberg, 1987, formerly *Pseudokirchneriella subcapitata* and *Selenastrum capricornutum*, is a standard test organism in ecotoxicology^22^. It is a fast-growing species that is sensitive to light and nutrients, and thus particularly well suited to evaluate stress effects on algal growth and production^23^. Algal culture for inoculation was grown for one week in Z8 media, with shaking (100–125 rpm) at room temperature and illumination of ~40 μE·m^−2^·s^−1^. Algal concentrations were determined using a 10 AU™ Field Fluorometer (Turner Designs, Sunnyvale, California, US).

### Chemicals and solutions

The chemicals were purchased from Sigma-Aldrich (Germany), and ^15^N-labeled NaNO_3_ was supplied by Cambridge Isotope Laboratories Inc. (USA). Isotopic composition of the non-labeled NaNO_3_ was determined with an elemental analyzer (EA) (Flash EA1112) coupled to the isotope ratio mass spectrometer (IRMS) (Thermo Delta + Advantage) via ConFlo III interface.

The culture media for algae contained: NaNO_3_ (6 mM), CaCl_2_·2H_2_O (73 mg/L), MgSO_3_·7H_2_O (25 mg/L), K_2_HPO_4_ (31 mg/L), Na_2_CO_3_ (21 mg/L), FeCl_3_·6H_2_O (28 mg/L) 0.001 M HCl, ZnSO_4_·7H_2_O (5 μg/L), MnCl_2_·4H_2_O (10 μg/L), H_3_BO_3_ (5 μg/L), CuSO_4_·5H_2_O (0.5 μg/L), CoCl_2_·6H_2_O (2 μg/L), NaMoO_4_·2H_2_O (1.5 μg/L), VOSO_4_·2H_2_O (0.5 μg/L), Na_2_SeO_4_·10H_2_O (0.5 μg/L), pH adjusted to 6.6–7.0^24^. In total, five experimental media with different at% ^15^N were prepared by mixing Na^15^NO_3_ and non-labeled NaNO_3_ stock solutions and used as treatments where 0.37 and 3.5 at% ^15^N represented resonance conditions, and 2, 10 and 50 at% ^15^N were the off-resonance conditions (Table 1).

### Experimental setup

A standard growth inhibition assay with *R. subcapitata* was conducted in microplate format. The assay is based on measurement of *in vivo* chlorophyll a (Chl *a*) that exhibits endogenous red fluorescence (autofluorescence). The quantification of chlorophyll fluorescence is useful for monitoring photosynthetic capacity and detection of stimulating or inhibiting effects. The algae were harvested and diluted with incomplete Z8 media, without nitrogen source to 5 × 10^5^ cell mL^−1^ and then mixed with the experimental media to the final cell density of 10^4^ cells ml^−1^. These suspensions were transferred to black-walled, clear bottom, 96-well plates (200 μl well^−1^) that were sealed to minimize evaporation. To avoid well-to-well contamination, we used one treatment per plate, three microplates per treatment, each with 88 wells for the test medium with algae and eight blank wells (treatment-specific medium without algae). All plates were incubated at room temperature and fluorescent light at 140 ± 10 μE·m^−2^ s^−1^. Light intensity was measured in the exposure area using photosynthetically active radiation sensor Quantum (Skye, UK). The algae were allowed to grow throughout their exponential period for 119 h without continuous shaking; the plates were shaken at least once a day in concert with fluorescence measurements. Fluorescence was measured at time points 0, 3, 21, 27, 45, 51, 69, 75, 97, 119 h using FLUOstar Optima microplate reader (BMG Labtech, Germany) with 640/400 nm for excitation/emission, ten measurements per well. The mean fluorescence of the plate-specific blanks was subtracted from each replicate. Over the range of cell densities used in this experiment, the fluorescence was linearly related to the cell number.

### Data analysis

The growth response of the algae exposed to each of the at% ^15^N concentrations was inferred from the measurements of chlorophyll fluorescence. Growth kinetics of the algae was examined using time-specific measurements fitted to an exponential growth curve with lag phase^25^. Using DMFit software (www.combase.cc), the lag phase duration (λ, h) and maximal growth during exponential phase (μ, d^−1^) for well-specific growth curves were estimated. The lag phase duration reveals how fast test organisms acclimate to specific conditions, while the growth rate in the exponential phase indicates proliferation in the adapted population (See Supplementary Information). Model fit was evaluated by the coefficient of determination (R^2^) and performance by the root mean square error (RMSE). An additional parameter, the change in fluorescence intensity between the observations (raw data) was used to calculate the area under the curve (AUC). This endpoint is commonly used in algal growth inhibition assay as it integrates the change in photosynthetically active biomass during both the lag and growth phases^26^.

### Statistics

When evaluating well-to-well variability in fluorescence, the coefficient of variation (CV%, n = 88) for each plate was calculated. To evaluate time and concentration effects on CV% as well as their interaction, we applied general linear model (GLM) with log link function and normal error structure. When a significant interaction was detected, the covariates were centered for evaluating the main effects^27^.

Because individual plates were assigned to different treatments, and the variance was unequal between the treatments (F test), we used a Restricted Maximum Likelihood Unequal-Variance (REML) mixed model with random intercepts to decompose the variances and derive parameter estimates. Using REML, we estimated the variance in the growth parameters (λ, μ, and AUC) due to one random effect—plate, and one fixed effect—treatment (at% ^15^N concentration). The random effect was always retained in the model while evaluating the fixed effect because the plates were part of the experimental design as a means to capture environmental variation due to possible differences in the light level, inoculum size and background fluorescence, rather than an explicit focus of the study. Testing whether the plates were significantly different from each other was irrelevant in this case. When significant treatment effects were detected, LSD pairwise comparisons (α = 0.05) were used to elucidate the degree to which individual treatment groups were different from each other; all tests were two-tailed. For each well-specific growth curve, REML was also used to evaluate the effect of ^15^N at% on the relationship between growth rate and lag phase, with μ as a dependent variable and λ and ^15^N as fixed factors and *plate* as a random factor. All endpoints were Box-Cox transformed to improve the residual structure of the models. The analyses were performed in S-PLUS 8.0 (TIBCO Software Inc.). Descriptive statistics are given as means and standard deviations unless otherwise stated.

## Results

**Figure 1:**
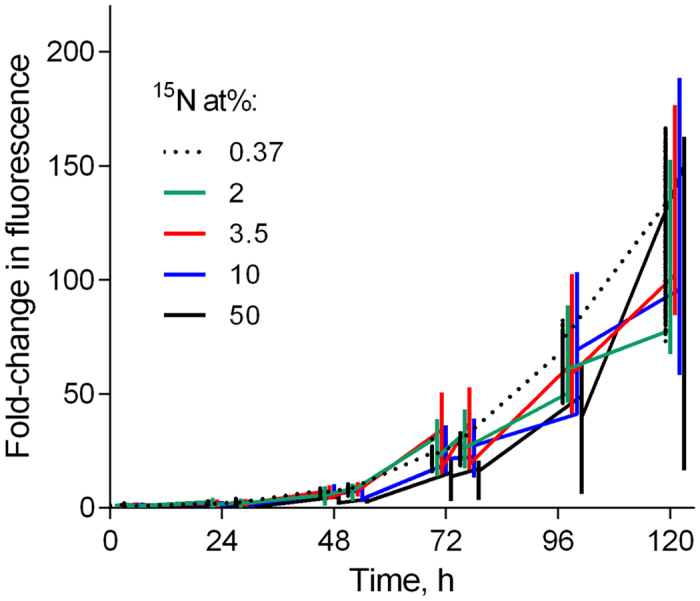
Fluorescence fold-change of *R. subcapitata* exposed to 0.37 to 50 at% ^15^N in the 119-h experiment. Primary observations for each well (median values with minimum and maximum; 88 wells per plate, three plates per treatment) are shown.

**Figure 2:**
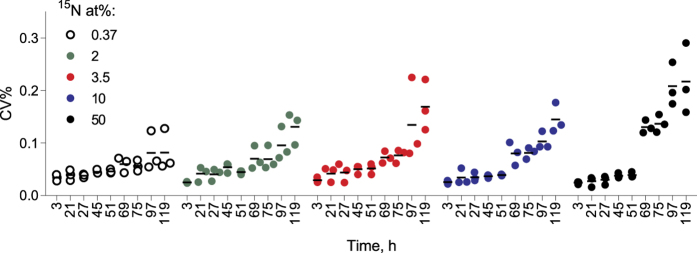
Change in coefficient of variation (CV%) of fluorescence values between wells for each plate (n = 88) grouped by treatment (^15^N at%; n = 3) over the course of the experiment; see Table2 for statistical evaluation.

Positive growth was observed in all treatments during the exposure, albeit with different growth trajectories (Fig. 1). Notably, the within-plate variability increased significantly with time and ^15^N at% concentration (Table 2; Fig. 2), with a coefficient of variation (CV%) reaching at the end of the incubation 8% and 22% in 0.37 and 50 at% ^15^N, respectively. Moreover, the time × concentration interaction effect on CV% was significant (Table 2), indicating that concentration effect became more pronounced with time.

**Figure 3:**
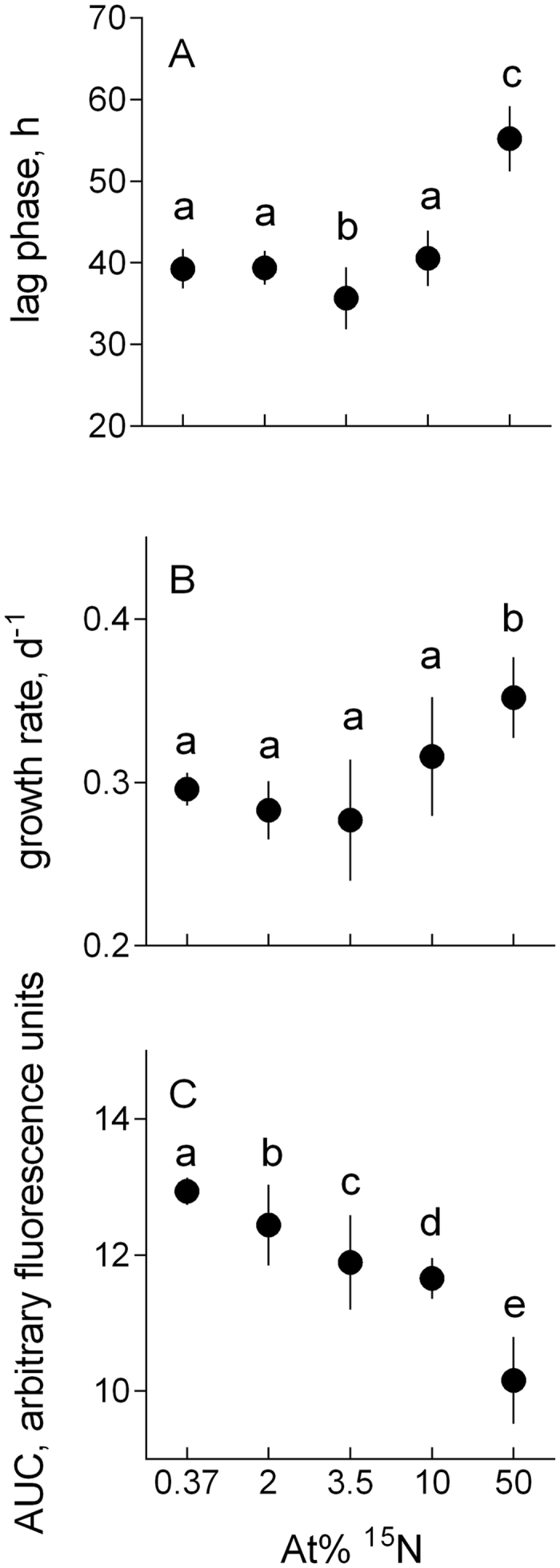
Kinetic parameters of growth responses in *R. subcapitata* exposed to 0.37 to 50 at% ^15^N: (**A**) Lag phase (λ) preceding the active growth, (**B**) maximal growth rate (μ) estimated for the exponential period, and (**C**) area under curve (AUC) representing algal production during the observation period. Since the *treatment* effect (^15^N at%) for λ, μ and AUC was significant regardless of the *plate* effect, the data are pooled within a treatment. Non-matching letters indicate a significant treatment difference at p < 0.05 evaluated by the REML procedure with *plate* as random factor and *treatment* as a fixed factor followed by the LSD multiple comparisons test; see Table 3B for the details. For illustrative purposes, data are shown as a grand mean for a treatment and SD (*n* = 3).

Both λ and μ values varied among the treatments, with particular deviations observed in 3.5 at% and 50 at% (Table 1, Fig. 3). High R^2^ values (>0.99 in all cases, with a few exceptions where it was >0.98) were observed for all well-specific growth curves. The lag phase significantly increased in algae exposed to 50 at% ^15^N (Fig. 3A) and significantly decreased in 3.5 at% ^15^N compared to that in the ambient (0.37) at% ^15^N concentration. From 0.37 to 3.5 at% ^15^N, the exponential growth rate decreased but then increased at the higher ^15^N concentrations, with significantly higher values at 50 than 0.37 at% ^15^N (Fig. 3B). The AUC values decreased monotonously with increasing at% ^15^N, each treatment being significantly different from all others (Fig. 3C).

**Figure 4:**
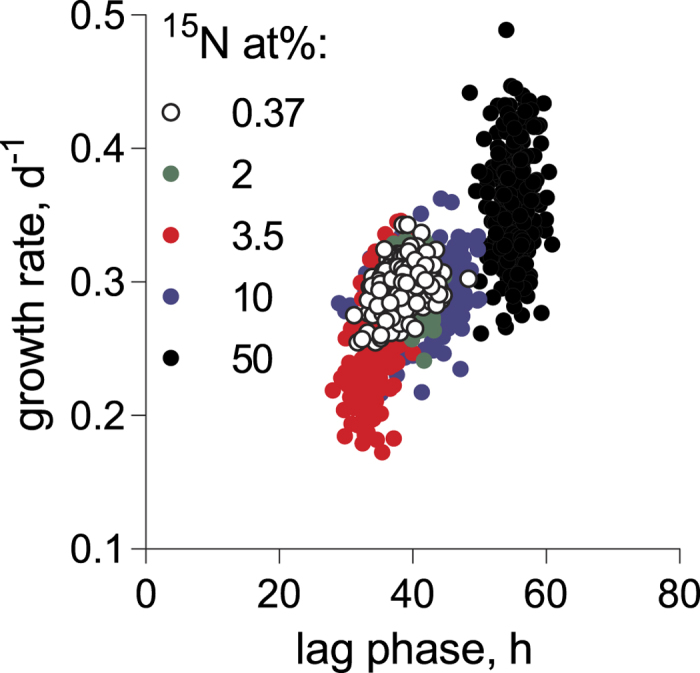
Relationship between maximal growth rate (μ, d^−1^) and duration of the lag phase (λ, h) in *R. subcapitata* exposed to 0.37 to 50 at% ^15^N.

There was an overall positive relationship between the maximal growth rate and lag phase duration across the treatments (Table 4A; Fig. 4). However, algae exposed to 50 at% ^15^N were deviating significantly from this relationship (Table 4B), because there was no significant relationship between the lag phase and growth within this treatment (GLM; R^2^ = 0.01, F ratio = 1.86, p > 0.2). Notably, the strongest and the most significant μ-λ relationship was observed in the 3.5 at% ^15^N treatment (GLM; R^2^ = 0.36, F ratio = 145.28, p < 0.0001).

## Discussion

Using standard growth inhibition assay, we observed significant effects of ^15^N enrichment on growth kinetics in eukaryotic algae. Compared to the 0.37 at% ^15^N, which represents ambient terrestrial ^15^N concentrations, all other treatments (2 to 50 at% ^15^N) have shown deviations in at least one growth parameter and the overall decline in productivity (Fig. 3). Since the total nitrogen supply was identical among the treatments, the observed effects can be attributed exclusively to the differences in the ^14^N/^15^N ratio in the nitrogen pool. These findings support the existence of KIEs in ^15^N-enriched algae (and, perhaps, other autotrophs), similar to what has been reported for bacteria *Escherichia coli* grown in media fully substituted with ^15^N^7^. In *E. coli* the ^15^N enrichment was found to affect key biosynthetic and metabolic pathways, such as citric acid cycle (a key metabolic pathway that unifies carbohydrate, fat, and protein metabolism), pyruvate production and glycolysis. These effects were observed in concert with growth inhibition and indicate changes in energy pathways^7^, which are highly evolutionary conserved; hence, it is not particularly surprising that algae growing on the ^15^N-enriched substrate were also affected. The mechanisms and magnitude of these KIEs as well as capacity to adapt would, however, vary depending on a taxonomic group and growth conditions, which needs to be tested and considered in studies involving isotope labeling.

We also found support for the non-monotonous response to ^15^N concentration in the growth medium, because the response of algae grown in 3.5 at% ^15^N deviated from the overall trends across the ^15^N concentration range. Whereas the monotonous decline by ~25% was observed for the total algal yield (AUC), the responses of the specific growth curve parameters were biphasic (Fig. 3), indicating possible differences in the adaptation depending on the isotopic composition of the media. In the algae grown at 3.5 at% ^15^N, the lag phase was reduced by ~10% and prolonged by > 40% in those grown at the highest ^15^N concentration (Fig. 3A). The response in maximal growth rate mirrored that of the lag phase, with 7% decline (albeit only marginally significant; Table 3B) and 19% increase in the 3.5 and 50 at% ^15^N treatments, respectively (Fig. 3B). These changes were greater or comparable to those observed in other treatments (0.37, 2.5, and 10 at%). The increase of growth rate in highly enriched media suggests that given sufficient time, the algae might fully adapt to the high ^15^N substrate, although in the time frame of this experiment, the total algal production was lower in the enriched media as indicated by the differences in the AUC values.[Table t4]

An immediate drop in fitness is commonly observed in microorganisms transferred to a stressful environment^28^. In algae, enzymatic NO_3_^−^ uptake follows Michaelis – Menten kinetics and thus should be sensitive to whether light or heavy isotope is taken up against gradient^29^. The isotopic discrimination is a function of both nitrogen availability and the isotopic composition of the substrate^30^,^31^. Recently, the ^15^N discrimination by nitrate reductase catalyzing the reduction of nitrate (NO_3_^−^) to nitrite (NO_2_^−^) in plants, fungi and bacteria has been reported to deviate significantly from the expected values when provided ^15^N enriched substrate; the authors have strongly advised against using highly enriched traces when studying nitrogen flows in plants or soils^31^. We concur with this recommendation and suggest that any concentration ≥2 at% ^15^N is sufficient to introduce alterations in algal metabolism. Moreover, Carliste *et al*.^31^ have demonstrated that experiments with enriched ^15^N tracers consistently produce lower estimates for N cycling rates than those conducted using natural abundance or non-isotopic methods. These observations, as well as our results, can be related to a phenomenon known as “isotope shock” – a prolonged lag phase in organisms introduced into the isotopically enriched environment. However, if nitrogen uptake and processing by the algal cells were progressively limiting with increasing ^15^N concentration due to KIE, the lag phase would increase monotonously from 0.37 to 50 at% ^15^N. This, however, was not the case as in 3.5 at% ^15^N the algae had the shortest lag phase of all treatments (Fig. 3A) indicating that adaptation occurred at a faster rate than in the 0.37 at% ^15^N; the latter represents an adaptation to the experimental conditions alone. Hence, an isotopic milieu with 3.5 at% ^15^N can be considered as beneficial for the short-term adaptation of the algae, which provides partial support for the IsoRes hypothesis. Notably, the maximal growth rates in the 3.5 at% ^15^N-adapted populations, i.e., following the lag phase, were the lowest (Fig. 3B), resulting in the intermediate total yield (Fig. 3C). Thus, different growth traits were not optimized simultaneously in this resonance conditions, at least within the time frame of our experiment.

In line with the treatment-specific growth and lag phase responses, there was a significant positive relationship between the growth rate in the adapted populations, and the lag phase over the range of ^15^N concentration tested (Fig. 4), indicating a coordinated response to the high ^15^N of the substrate. Moreover, in the algae exposed to the highest ^15^N concentration, this relationship was not pronounced, most likely due to the low variability of the lag time, but also, perhaps, because of the different trade-offs regulating adaptability in this isotopic environment. We are not aware of any studies addressing relationships between the growth phases in algae under stress. The prevailing view for other microorganisms is that during the lag phase cells are adapting to the new environment, and cell division is arrested^32^,^33^,^34^,^35^. However, these adaptations are poorly understood due to the low metabolic rate of such cells and usually the insufficient amount of material for analysis. It has been suggested that during the lag phase, bacterial cells are preparing for maximal growth upon transition to the exponential phase^32^. In some cases, lag phase extension is associated with increased invasiveness of bacterial strains^33^, fighting pathogens^33^, or with antibiotic tolerance^34^. Evolutionarily, gene expression is shaped to prepare a cell for growth burst^35^; hence, it is possible that in the lag phase, the metabolome of organisms exposed to unfamiliar isotope conditions is adjusting to the kinetic equilibrium. Also, selection for a growth genotype that is likely to occur during the prolonged lag phase would result in cell populations that have a higher capacity to use ^15^N compared to the inoculated culture. Therefore, it is possible that total yield of well-adapted algae exposed to elevated ^15^N concentrations during longer time periods will increase due to the higher growth rates that are potentially attainable; this, however, remains to be tested.

More experimental studies are needed to look into the processes and responses in the short and long run and to reveal what characteristics under what conditions confer selective advantage in various organism groups. The differences between taxa can depend on N uptake mechanisms employed and non-stationary isotopic effects because of competitive or synergistic processes that affect the kinetics of targeted compounds as well as the chemical form of the substrate used. Some processes, such as microbial nitrite oxidation where an inverse kinetic isotope effect on fractionation has been found^36^ may benefit from ^15^N increase in the substrate. To predict growth, metabolism and biochemical composition responses, it is necessary to know the isotopic imprints characteristic of respective N transformations, to understand the underlying mechanisms that determine these patterns, and to link them to the downstream processes of energy and material cycling.

For stable isotope applications in environmental microbiology, it is also relevant to investigate how a pulse addition of the label to a population that is either actively growing or in a steady-state may influence organism growth and metabolism, and what effects are most likely to occur during a standard incubation duration (24–48 h). As suggested by our results, the label addition may result in growth inhibition. Moreover, the response would be different compared to the effects on the maximal growth rates observed in our study, because during the lag phase our algae had a possibility to adjust and, thus showed a higher growth potential during the exponential phase. In the absence of the adaptation, growth will be inhibited for a period comparable with the lag phase (i.e., 35–40 h for green algae similar to those used in our experiment). After this time, the algal growth response to ^15^N would resemble what we have observed in the adapted population (Fig. 3B).

Another area of concern with respect to the adaptation to the elevated ^15^N levels is the increase of within- (Figs 2 and 3) and between-plate (Fig. 3) variability for all measured endpoints. For various physiological responses, both individual and population variances often increase under stress^37^. Moreover, higher variability of not only lag times but also their particular distributions within a population can be induced by various stressors^38^. Thus, the observed ^15^N effect on CV% for population abundance (Table 2), and the underlying variability of lag phase and growth rate among the metapopulations in different wells (Figs 1 and 3) are indicative of the stressful conditions. Various adaptation strategies exploiting individual behavior of algal cells during the exposure and, particularly, during the lag phase, may exist. The mechanisms involved in physiological adaptations that allow to counteract KIEs and maintain high growth are at present unknown, but one can speculate that it may involve alterations in nitrogen uptake, metabolism, and bioenergetics. Also, selection of cells that rely on heavy-isotope specific enzymes^36^,^39^, thus improving the population capacity to use heavier isotopes, would be likely.

In ecological studies, enrichment of stable isotopes as tracers is a standard approach to study element flows and trophic interactions occurring within food webs. Various methods have been developed to enrich bacteria, algae, detritus and plant litter, earthworms, crustaceans, and other food web components to trace the passage of ^13^C and ^15^N through ecosystems 1. The amount of ^15^N and ^13^C label employed in these methods and experiments is usually around 2–35 at%^40^,^41^. However, even much higher values are not rare^42^, up to the full replacement of an element by its heavy isotope in DNA^43^ and proteome^44^ labeling. None of the terrestrial organisms are evolutionary suited to such substrates, because during the elemental and isotope evolution of the planet surface δ^15^N never exceeded 35‰. The latter occurred around 2.7 Ga and, when the first eukaryotic algae have appeared, δ^15^N values have stabilized at about 5‰^45^. The severe reduction of the total yield (up to 23%) and the observed changes in growth strategies of the algae exposed to 2 to 50 at% ^15^N (δ^15^N > 4495‰) and prokaryotes grown on isotopically enriched medium^7^,^17^ suggest that current way of applying isotope labeling may induce significant alterations in biomass production and material transfer in various microorganisms. It also raises a question whether similar effects occur in eukaryotic heterotrophs, including larger consumers, as a result of stable isotope labeling. The responses observed suggest that the assumption of no effect generated by the label incorporation itself may not hold true, with far-reaching implications for the interpretation of ecological effects addressed in such experiments. Moreover, the increased variance in the isotope-labeled systems should make detection and interpretation of the effects in question more difficult.

## Additional Information

**How to cite this article:** Andriukonis, E. and Gorokhova, E. Kinetic ^15^N-isotope effects on algal growth. *Sci. Rep.* **7**, 44181; doi: 10.1038/srep44181 (2017).

**Publisher's note:** Springer Nature remains neutral with regard to jurisdictional claims in published maps and institutional affiliations.

## Supplementary Material

Supplementary Information

## Figures and Tables

**Table 1:** Target ^15^N concentrations (at%) in the experimental growth media and corresponding proportions of Na^15^NO_3_ (98 at% ^15^N; Cambridge Isotope Laboratories Inc., USA) and non-labeled NaNO_3_ (0.37004 at%; own measurements) stock solutions used to generate the experimental concentrations.

Compound	0.37 at%	2 at%	3.5 at%	10 at%	50 at%
Na^15^NO_3_	0.0000	0.0167	0.0321	0.0986	0.5083
non-labeled NaNO_3_	1.0000	0.9833	0.9679	0.9014	0.4917

**Table 2:** GLM output testing effects of time and ^15^N concentrations (at%) in the experimental growth media on the well-to-well variability of algal fluorescence expressed as coefficient of variation (CV%, dependent variable) within a plate.

Independent variables	MS	F	*p*
Time	0.033	155.25	**<0.0001**
^15^N	0.003	11.05	**0.0012**
Time × ^15^N	0.010	46.31	**<0.0001**

Three plates per treatment with 88 wells per plate were used; R^2^_adj._ = 0.78.

**Table 3:** REML output for (A) models testing effects of ^15^N concentration (at%; fixed effect) on the lag phase duration (λ, h), maximal growth during exponential phase (μ, d^−1^) and AUC for well-specific growth curves over 119 h in the test system. *Plate* was a random effect in each model, and (B) LSD pairwise comparisons. Significant p-values are indicated in bold.

**A**)					
Dependent variable	Num df	Den df	F ratio	p-value	
Lag phase, λ	3	8	48.27	**<0.000**	
Maximal growth rate, μ	4	10	16.17	**0.0002**	
AUC	4	10	219.04	**<0.000**	
**B**)					
**Dependent variable**	**Treatment groups**	**2 at%**	**3.5 at%**	**10 at%**	**50 at%**
Lag phase, λ	0.37 at%	0.920	**0.024**	0.359	**<0.000**
	2 at%		**0.020**	0.411	**<0.000**
	3.5 at%			**0.004**	**<0.000**
	10 at%				**<0.000**
Maximal growth rate, μ	0.37 at%	0.433	0.072	0.538	**0.001**
	2 at%		0.261	0.862	**0.001**
	3.5 at%			0.201	**<0.000**
	10 at%				**0.001**
AUC	0.37 at%	**0.001**	**<0.000**	**<0.000**	**<0.000**
	2 at%		**0.001**	**<0.000**	**<0.000**
	3.5 at%			**0.031**	**<0.000**
	10 at%				**<0.000**

**Table 4 t4:** REML output for (A) the model testing effects of ^15^N concentration (at%; fixed effect) and the lag phase duration (λ, h) on the maximal growth during exponential phase (μ, d^−1^); data are matched by well (Fig. 4) and *Plate* was a random effect in each model, and (B) probabilities for significant differences between the treatment groups evaluated by LSD pairwise comparisons.

**A**)				
Fixed factors	Num df	Den df	F ratio	p-value
Lag phase, λ	1	1305	88.89	**<0.000**
^15^N concentration	4	10	6.87	**0.006**
**B**)				
**Treatment groups**	**2 at%**	**3.5 at%**	**10 at%**	**50 at%**
0.37 at%	0.367	0.107	0.389	**0.007**
2 at%		0.423	0.966	**0.002**
3.5 at%			0.402	**0.001**
10 at%				**0.002**

Significant p-values are indicated in bold.
