# Supplementary material for: Kinetic 15N-isotope effects on algal growth
Source: Sci Rep. 2017 Mar 10;7:44181. doi: 10.1038/srep44181 (PMC5345060; doi:10.1038/srep44181)
Supplement: Supplementary Information [file srep44181-s1.pdf]

Supplementary Information

Kinetic ^{15}N-isotope effects on algal growth

Eivydas Andriukonis and Elena Gorokhova

Algal growth model

Similar to bacterial growth, algal growth model often consists of several phases following a logistic or sigmoid curve. The lag period duration (λ), the maximum specific growth rate (μ_{max}) and the asymptotic value (A) are the most informative parameters of this curve (Fig. S1). When algal cells are inoculated into a limited volume of culture medium and exposed to suitable conditions of light, nutrients, and temperature, there is a lag phase, an exponential phase, a phase of declining relative growth rate and a stationary phase. During the lag phase, cell protein and nucleic acid contents increase and we may surmise that this phase is one of reconstitution, in which enzyme and substrate concentrations are built up to the degrees necessary for multiplication. In the exponential phase, the organisms have a high capacity for photosynthesis, and the products are used mainly for the synthesis of protein. In a static culture, the exponential phase ends after a time because of depletion of nutrients, accumulation of toxic by-products of metabolism, or simply because light becomes limiting as the culture becomes denser (Fogg 1957).

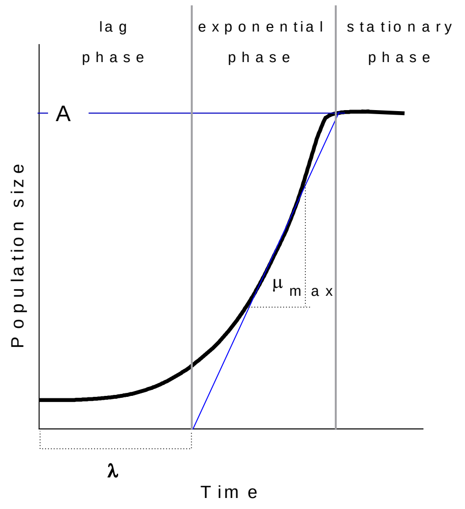

Fig. S1. Schematic diagram representing temporal development of algal population size that follows main phases of the growth relevant for the study: lag phase and exponential increase that levels off reaching a stationary phase. Duration of the lag phase is denoted as λ, and μ_{max} is maximal growth rate value. The lag phase just after $t = 0$ is characterized by zero growth; it is followed by the exponential phase with acceleration to a maximal value (μ_{max}) and, finally, a stationary phase with zero growth.

The Baranyi-Roberts model (Baranyi and Roberts 1994) based on a modified logistic equation was chosen to fit the growth profile of the algae under various ^{15}N levels. This model is commonly

reported to produce a good fit for bacterial growth curves (Coleman et al. 2003; López et al. 2004), especially with varying lag phase (McKellar and Knight 2000; Pin et al. 2002; Baty et al. 2004; Fujikawa et al. 2004). In addition to the good fitting capacity for many microorganisms, the model is popular because most of the model parameters being biologically interpretable (McKellar and Knight 2000; López et al. 2004; Van Impe et al. 2005). The Baranyi-Roberts model has been successfully used to model algae growth in culture (Lacerda et al. 2011; Tevatia et al. 2012; Halmi et al. 2014; Mohamed et al. 2014). Moreover, recent evaluations suggest its superiority in microorganism growth modeling (Van Impe et al. 2005), including algae (Halmi et al. 2014) under stress and limiting conditions.

The model is based on the first-order differential equation predicting specific growth rate, μ(t) (h^{-1}) of the cell population with time (Baranyi et al., 1993; Baranyi, 1997):

$$\mu(t) = \frac{1}{X(t)}\frac{dX}{dt} = \mu_{\max}\alpha'(t)f(t) \qquad (1)$$

where *X*(t) is the algal concentration in the medium at time *t* (expressed as total fluorescence at constant culture volume), $\mu_{\max}$ is the maximum specific growth rate (d^{-1}), *α*'(*t*) is the adjustment function, and *f*(*t*) is the inhibition function.

Further, *α*'(*t*) in the Eq. (1) is a monotonously increasing function, being $0 \leq \alpha'(t) \leq 1$ and $lim_{t\to\infty}\alpha'(t) = 1$ describes the adaptation of algal cells when entered in the new environment of the test conditions, i.e. during the lag phase. It is based on a kinetic assumption that the growth of cells in the lag phase is inhibited by a limiting intracellular substance following a Michaelis–Menten principle (Baranyi and Roberts, 1994; Baranyi, 1997) and expressed as (Perni et al., 2005):

$$\alpha'(t) = \frac{e^{-h_0}}{e^{-\mu_{\max}t} + e^{-h_0} - e^{-\mu_{\max}t-h_0}} \qquad (2)$$

where h_0 is the dimensionless Baranyi-Roberts model parameter.

Finally, *f*(t) in the Eq. (1) is a monotonous decreasing function with *f*(0) = 1 and $lim_{t\to\infty}\alpha'(t) = 1$, which is described by the following logistic function:

$$f(t) = 1 - \frac{X}{X_{max}} \tag{3}$$

where X_{max} is maximal algal concentration observed in during the exposure and represented by the total fluorescence at constant test volume.

After rearranging Eqs. (1)–(3), the rate of microalgae growth (dX/dt) becomes:

$$\frac{dX}{dt} = \mu_{max}\left(\frac{e^{-h_0}}{e^{-\mu_{max}t} + e^{-h_0} - e^{-\mu_{max}t-h_0}}\right)\left(1 - \frac{X}{X_{max}}\right) \tag{4}$$

The cell population in a batch reactor at time t is modeled by integrating Eq. (4) at initial conditions: $X(0) = X0$ and $X(t) = X(t)$:

$$X(t) = \frac{X_{max}X_0(e^{h_0} + e^{\mu_{max}t} - 1)}{X_{max}e^{h_0} + X_0(e^{\mu_{max}t} - 1)} \tag{4}$$

Individual (i.e. well-specific) growth curves fitted to the fluorescence measured on each observation occasion were produced to calculate μ_{max} and duration of the lag phase. For these calculations, DMFit software (*www.combase.cc*) was used applying models with no asymptote when cell population had not reached a stationary phase or a complete model when the stationary phase was detected. Altogether, about 50% of the metapopulations (wells) have reached the stationary phase during the exposure.

References

Baranyi J 1997. Simple is good as long as it is enough. Food Microbiol 14, 189–192.

Baranyi J, Roberts TA, McClure P. 1993. A non-autonomous differential equation to model bacterial growth. Food Microbiol 10, 43–59.

Baranyi J, Roberts TA. 1994. A dynamic approach to predicting bacterial growth in food. Int J Food Microbiol 23(3-4):277–94.

Baty F, Delignette-Muller M-L. 2004. Estimating the bacterial lag time: Which model, which precision? Int J Food Microbiol 91(3): 261–77.

Coleman ME, Tamplin ML, Phillips JG, Marmer BS. 2003. Influence of agitation, inoculum density, pH, and strain on the growth parameters of *Escherichia coli* O157:H7 - Relevance to risk assessment. Int J Food Microbiol 83(2): 147–60.

Fujikawa H, Kai A, Morozumi S. 2004. Improvement of new logistic model for bacterial growth. J Food Hyg Soc Jpn 45(5): 250–254.

Fogg GE (1957) Relationships between Metabolism and Growth in Plankton Algae. J Gen Microbiol 16: 294-297.

Halmi MIE, Shukor MS, Johari WLW, Shukor MY (2014) Evaluation of several mathematical models for fitting the growth of the algae *Dunaliella tertiolecta*. Asian J Plant Biol 2: 1-6.

Lacerda LMCF, Queiroz MI, Furlan LT, Lauro MJ, Modenesi K, Jacob-Lopes E, et al. 2011. Improving refinery wastewater for microalgal biomass production and CO_2 biofixation: Predictive modeling and simulation. J Pet Sci Eng 78(3-4): 679–86.

López S, Prieto M, Dijkstra J, Dhanoa MS, France J. 2004. Statistical evaluation of mathematical models for microbial growth. Int J Food Microbiol 96(3): 289–300.

McKellar RC, Knight K. 2000. A combined discrete-continuous model describing the lag phase of *Listeria monocytogenes*. Int J Food Microbiol 54(3):171–80.

Mohamed MS, Tan JS, Kadkhodaei S, Mohamad R, Mokhtar MN, Ariff AB. 2014. Kinetics and modeling of microalga *Tetraselmis* sp. FTC 209 growth with respect to its adaptation toward different trophic conditions. Biochem Eng J 88: 30–41.

Perni S, Andrew PW, Shama G. 2005. Estimating the maximum growth rate from microbial growth curves: definition is everything. Food Microbiol 22: 491–495.

Pin C, García de Fernando GD, Ordóez JA, Baranyi J. 2002. Analysing the lag-growth rate relationship of *Yersinia enterocolitica*. Int J Food Microbiol 73(2-3): 197–201.

Tevatia R, Demirel Y, Blum P. 2012. Kinetic modeling of photoautotropic growth and neutral lipid accumulation in terms of ammonium concentration in *Chlamydomonas reinhardtii*. Biores Technol 119:419–24.

Van Impe JF, Poschet F, Geeraerd AH, Vereecken KM. 2005. Towards a novel class of predictive microbial growth models. Int J Food Microbiol 100(1-3): 97–105.
